# Beyond coexistence: the asymmetric interplay of health risk and promotion pathways driven by communication legacies across emerging and recurring epidemics

**DOI:** 10.3389/fpubh.2026.1835961

**Published:** 2026-04-24

**Authors:** Shaolong Wu, Haiyan Wan

**Affiliations:** 1 School of Life Sciences, Central South University, Changsha, China; 2 Xiangjiang New Area Administrative Committee, Changsha, China

**Keywords:** asymmetric interplay, emergency risk communication, emerging epidemics, health behavior, psychological legacy, recurring epidemics

## Abstract

**Objective:**

Prior emergency risk communication incidents create enduring psychological legacies that complicate the interplay between health-promoting and health-risk tendencies. This study investigates how negative and positive legacies jointly operate within this spillover effect. Specifically, we examine whether positive legacies can offset the adverse effects of negative ones and identify the boundary conditions moderated by emerging versus recurring epidemics—under which this buffering mechanism fails.

**Methods:**

Grounded in the Stress Spillover Model and Post-traumatic Growth Theory, a dual-path parallel mediation model for PERCI was constructed. Adopting a cross-sectional survey combined with scenario simulation to capture cross-epidemic effects, we recruited 822 participants who have experienced COVID-19 via convenience and snowball sampling. Data were examined using structural equation modeling.

**Results:**

PERCI exerts significant spillover effects via an asymmetric dual-path mechanism. While parallel mediation by both positive and negative legacies was confirmed, a crucial asymmetry emerged: the negative pathway substantially dominated the positive one, indicating that risk impulses often override promotional resources. However, boundary condition analysis revealed a divergent regulation: in recurring epidemics, positive legacies significantly strengthened the promotion pathway (*β* = 0.14, *p* < 0.05), effectively offsetting negative inertia. Conversely, in emerging epidemics, negative legacies reinforced the risk pathway (*β* = 0.15, *p* < 0.05), creating scenarios where risk behaviors remain largely unchallenged by positive resources.

**Conclusion:**

While negative legacies exert a dominant risk effect, positive legacies provide an independent protective pathway that partially offsets this inertia, but only under specific boundary conditions. These findings highlight a critical, non-linear interplay in cross-epidemic responses. Public health interventions must adopt a context-specific dual strategy: simultaneously mitigating negative legacies and strengthening positive ones. Crucially, standard promotional tactics may fail to counteract dominant risk pathways during emerging outbreaks, necessitating tailored communication strategies that account for the asymmetric nature of psychological legacies.

## Introduction

The global COVID-19 pandemic unprecedented in both duration and uncertainty (1, 2)—has highlighted a critical challenge in public health: the dynamic interplay and coexistence between health-promoting tendencies (PT) and health-risk behavioral tendencies (RT) (3). While effective emergency behavioral tendencies rely on the public’s adoption of protective measures, crises often simultaneously trigger maladaptive risk behaviors. Emerging evidence (4) suggests that these divergent behavioral trajectories are not merely reactions to immediate information but are deeply rooted in historical cognitive legacies accumulated from PERCI. Specifically, past interactions with health authorities and crisis management may leave enduring psychological residues that function as either cognitive resource for protection or susceptibility factors for risk. Yet, a pivotal question remains: How do these accumulated legacies differentially drive the competition between promoting and risking tendencies in future scenarios? More importantly, does the nature of the future threat—whether an emerging or a recurring epidemic—act as a boundary condition that shifts the balance between these opposing behavioral forces? Addressing this inquiry is essential for moving beyond static models of communication effects toward a dynamic understanding of how historical memory shapes the behavioral interplay in successive health crises.

Previous scholarship has firmly established that PERCI exert immediate impacts on public trust and compliance (4, 5). Extensive evidence confirms that adverse incidents, such as mishandled uncertainties (6) or ineffective rumor rebuttals (7), undermine public trust in health authorities. Several studies have begun to extend the temporal horizon, acknowledging that disaster experiences can transcend single crises; for instance, SARS exposure shaped trust dynamics during COVID-19 (8). However, despite these advancements, two critical gaps persist that hinder a comprehensive behavioral understanding. First, regarding effect valence, existing literature remains largely confined to a deficit-focused framework, overlooking the potential for positive experiences to accumulate as protective cognitive capital. It remains unclear how positive legacy (PL) and negative legacy (NL) coexist and compete to shape behavioral intentions, and whether PL can effectively offset the adverse inertia of NL. Second, regarding contextual granularity, current research treats epidemic contexts as homogeneous, failing to distinguish between emerging epidemics and recurring epidemics. Consequently, it is unknown whether the spillover mechanisms of PERCI operate symmetrically across these distinct scenarios, or if specific contexts exist where health-risk behavioral tendencies (RT) remain largely unchallenged by promoting efforts. This study addresses these gaps by proposing a Temporal-Contextual-Valence Framework, aiming to elucidate how cumulative micro-level PERCI evolve into bidirectional psychological legacy that drive asymmetric behavioral outcomes under varying epidemic conditions.

While individual PERCI may function as isolated stressors, their psychological impacts accumulate over time to form an enduring legacy that serves as a precursor to future health behaviors. Integrating the Stress Spillover Model (SSM) (9) with Post-traumatic Growth Theory (10), this study reconceptualizes PERCI not merely as emotional echoes, but as cumulative cognitive constructs that differentially prime health-promoting behavioral tendencies (PT) and RT. In this framework, PL acts as a resource facilitating protective actions, while NL functions as a susceptibility factor triggering maladaptive behavioral tendencies. This integrated approach yields two key theoretical contributions. First, we empirically validate a bidirectional mediation mechanism, demonstrating that PERCI shapes future behavioral tendencies through parallel pathways of PL and NL, thereby resolving the literature’s unidirectional bias. Second, we propose and test an Asymmetric Dual-Pathway Model moderated by Epidemic Context. We identify two forms of asymmetry: magnitude asymmetry, wherein the negative pathway dominates in emerging epidemics due to heightened uncertainty; and contextual asymmetry, where the positive pathway gains prominence in recurring epidemics as familiarity allows cognitive resources to overcome fatigue. Collectively, these findings advance our understanding of the behavioral interplay across successive crises, revealing specific contexts where risk tendencies resist counteracting forces. From a practical standpoint, this study informs staged intervention strategies: prioritizing the blocking of risk behavior chains in emerging epidemics while activating promoting resources in recurring scenarios.

## Theoretical framework and research hypotheses

### Key concept definitions

#### Prior emergency risk communication incidents

PERCI is reconceptualized not merely as a record of communication failures, but as a cumulative behavioral memory. It represents the aggregated subjective intensity of past interactions with health authorities during epidemics. As a historical stressor, PERCI forms the foundational schema that primes individuals’ susceptibility to risk behaviors or their capacity for protective actions in future crises.

#### Psychological legacy as cognitive precursors

##### Psychological legacy

Defined as Cognitive Resources for health-promoting behaviors tendenties. It encompasses internalized resilience, trust in scientific authority, and a perceived efficacy of collective action. PL serves as the psychological capital that facilitates adaptive behavioral tendencies.

##### Negative legacy (NL)

Defined as Susceptibility Factors for RT. It comprises solidified anxiety schemas, cynical attitudes toward authority, and avoidance patterns. NL acts as a latent trigger that predisposes individuals to maladaptive behavioral tendencies when threatened.

#### Epidemics context as boundary conditions of behavioral interplay

Epidemic Context refers to the immediate environmental background in which individuals make behavioral decisions. To address the ambiguity between emerging and recurring scenarios, this study strictly defines them based on pathogen familiarity and local epidemic history.

To elucidate the dynamic competition between promoting and risking tendencies, we strictly define two contextual boundary conditions:

Emerging Epidemic (High Uncertainty): A scenario involving an emerging epidemic with no local circulation history. Characterized by information ambiguity and high perceived threat, this context acts as a catalyst that preferentially activates risk behavioral inertia.

Recurring Epidemic (High Familiarity): A scenario involving a known pathogen with prior local exposure. Characterized by cognitive fatigue and experiential learning, this context allows promoting behavioral resources to gradually accumulate and compete against risk impulses.

#### Behavioral tendencies (proxies for actual behavior)

PT: Constructive intentions indicative of protective actions.

RT: Destructive or defensive intentions indicative of maladaptive actions.

### The temporal-contextual-valence framework

To systematically unpack these dynamics, we propose a novel Temporal-Contextual-Valence (TCV) Framework. This framework posits that health behavioral outcomes are not determined by single factors but emerge from the intersection of three critical dimensions:

The Valence Dimension (Historical Legacy): Representing the quality of past experiences, ranging from PL to NL.

The Contextual Dimension (Epidemics context): Representing the environmental boundary, distinguishing between the high-uncertainty Emerging context and the familiar Recurring context.

The Temporal-Behavioral Dimension (Response Orientation): Representing the directional outcome, contrasting immediate PT with RT.

Crucially, the TCV Framework argues that the Valence-Behavior link is not static; it is dynamically modulated by the Contextual Dimension. Specifically, we theorize that the Emerging context acts as a risk amplifier, strengthening the path from Negative Valence to Risk Behavior while weakening the path from Positive Valence to Promoting Behavior. Conversely, the Recurring context serves as a stability restorer, allowing Positive Valence to regain its predictive power. This three-dimensional interaction forms the theoretical basis for our hypotheses.

### Research hypotheses

This study adopts the SSM as its overarching framework, integrated with PTG and TPB. While individual traits matter (11, 12), Situation Strength Theory posits that in macro-level health crises, environmental cues (Emerging vs. Recurring) exert a dominant influence on the activation of behavioral pathways. We focus on these contextual boundary conditions to explain the asymmetric interplay between PT and RT.

#### Main effect hypotheses

We propose a parallel dual-pathway model where PERCI bifurcates into distinct cognitive precursors:

*H1*: PERCI has a significant positive impact on PL.

*H2*: PERCI has a significant positive impact on NL.

*H3*: PL has a significant positive impact on PT.

*H4*: NL has a significant positive impact on RT.

*H5*: Although the mediated pathways are expected to dominate, we tentatively propose that PERCI may exert a residual direct impact on PT, reflecting heuristic processing in the absence of strong cognitive mediation.

*H6*: PERCI exerts a significant direct impact on RT, reflecting immediate threat-response heuristics that bypass cognitive appraisal.

#### Mediation hypotheses: the role of historical memory

PTG theory emphasizes that the long-term impact of stress is mediated by psychological reconstruction. We posit that psychological legacy serves as the critical mechanism translating historical communication experiences into current behavioral inclinations.

*H7*: PL mediates the relationship between PERCI and PT.

*H8*: NL mediates the relationship between PERCI and RT.

#### Moderation hypotheses: contextual asymmetry in behavioral interplay

The core contribution of this study lies in identifying how Epidemic Context alters the strength of these behavioral pathways. We draw on Negativity Bias Theory (13) and Cognitive Resource Models (14) to propose an asymmetric pattern.

The Dominance of Risk in Emerging Contexts: In emerging epidemics, high uncertainty triggers a primal threat response. According to Negativity Bias, NL weigh more heavily than positive ones under high threat. Consequently, the susceptibility to RT driven by NL should be amplified, potentially overriding promoting efforts. The fear of the unknown makes the risk pathway hyper-sensitive.

In recurring epidemics, familiarity reduces immediate panic, allowing cognitive resources (PL) to play a more substantial role. While fatigue exists, the accumulation of experience enables individuals to rely more on established trust and efficacy beliefs (PL) to guide behavior, making the promoting pathway relatively more potent compared to the chaotic emerging phase. Alternatively, the sheer exhaustion in recurring phases might dampen all active behavioral tendencies, but critically, the relative dominance of the risk pathway (NL linking to RT) observed in emerging phases should diminish, allowing the positive pathway to gain comparative traction.

Based on this theoretical reasoning, we propose the following revised moderation hypotheses:

*H9*: Epidemic Context moderates the relationship between PL and PT. Specifically, the positive impact of PL on PT is stronger in the Recurring epidemic context than in the Emerging epidemic context.

*H10*: Epidemic Context moderates the relationship between NL and RT. Specifically, the positive impact of NL on RT is significantly stronger in the Emerging epidemic context than in the Recurring epidemic context.

#### Summary of the theoretical model

**Figure 1:**
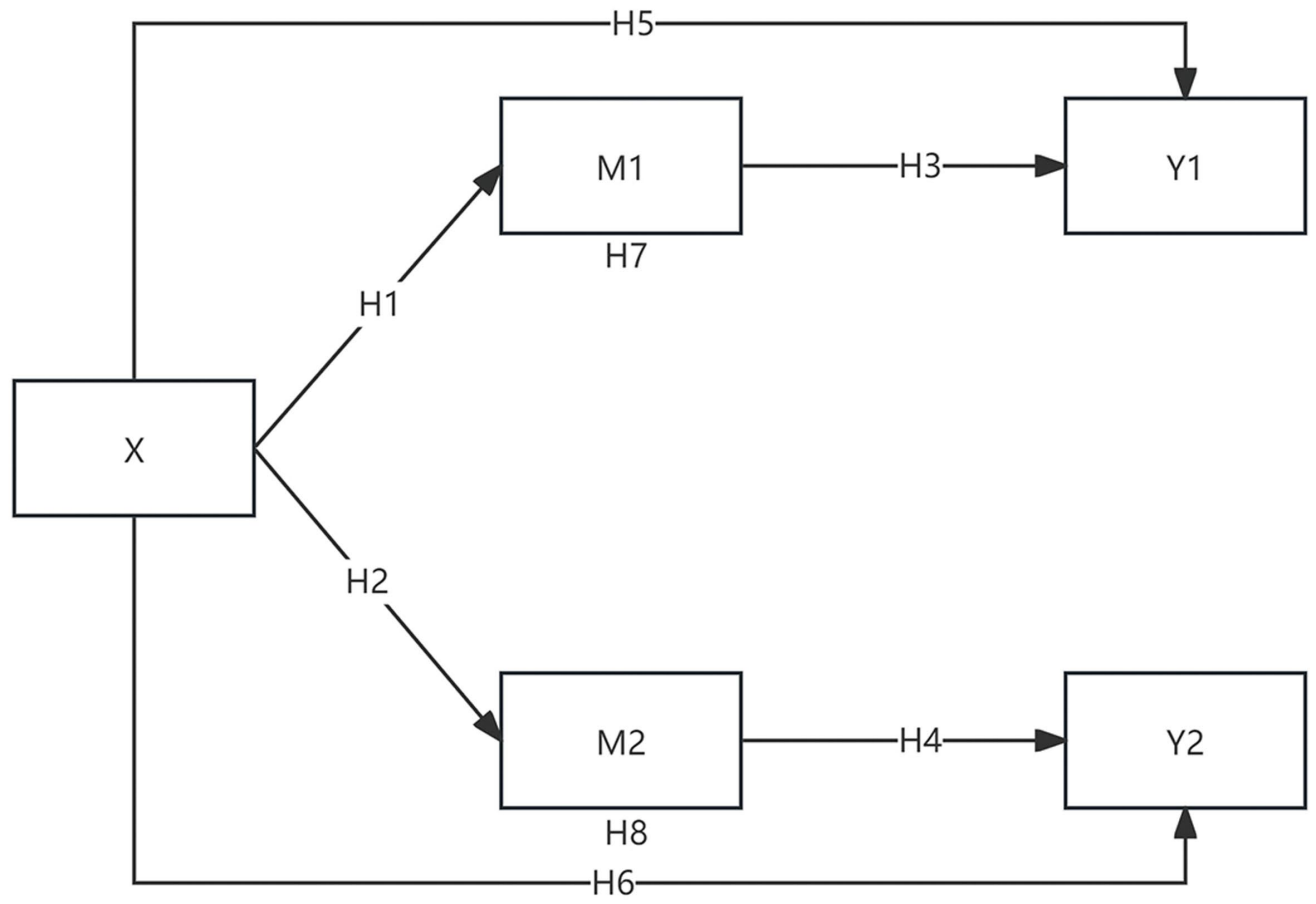
PERCI cross-epidemic spillover effect model. X: PERCI; Y1: health-promoting behavioral tendencies (PT); Y2: health-risk behavioral tendencies (RT); M1: positive psychological legacy (PL); M2: negative psychological legacy (NL). For clarity, potential moderators (situational factors) are not shown but are examined in subsequent analyses.

The theoretical model of this study, illustrating the main effects, mediation paths, and moderation boundaries, is presented in Figure 1.

## Methods

### Research design and data collection

You may insert up to 5 heading levels into your manuscript as can be seen in “Styles” tab of this template. These formatting styles are meant as a guide, as long as the heading levels are clear, Frontiers style will be applied during typesetting.

#### Research design

This study employed a mixed design combining a cross-sectional survey with a scenario-based behavioral simulation. The primary objective was to examine how historical cognitive legacies (accumulated from PERCI) interact with contextual boundary conditions to drive competing PT and RT.

Specifically, the cross-sectional component captured participants’ cumulative perceptions of PERCI and their resulting psychological legacy (PL and NL). Subsequently, a randomized scenario experiment manipulated the Epidemic Context (Emerging vs. Recurring) to activate distinct behavioral pathways. This design ensures high ecological validity regarding historical memory while maintaining the internal validity necessary to infer causal mechanisms in behavioral decision-making. The study protocol was approved by the Ethics Committee of the School of Life Sciences, Central South University (Approval No. CSU-LS-H-2026-1-2).

#### Sample size and power analysis

A two-stage data collection strategy was implemented:

Pilot Study: A sample of *N* = 237 was used to refine the core scales through item analysis and Exploratory Factor Analysis (EFA) (15). This sample size adheres to the empirical rule of having at least 5 times the number of scale items (16).

Formal Study: The target sample size was determined via comprehensive power analysis using G*Power 3.1 (17), Given the complexity of the Asymmetric Dual-Path Model, we aimed to detect small-to-medium effect sizes *f*^2^ = 0.045, *α* = 0.05, and power = 0.90, a minimum sample of 680 was required. Furthermore, based on the N: q rule for Structural Equation Modeling (SEM) with approximately 70 estimated parameters, a minimum of 700 was recommended. Consequently, we targeted a sample between 800 and 900. The final valid sample of *N* = 822 exceeded these thresholds, ensuring robust statistical power to test the hypothesized contextual asymmetries.

#### Data collection and screening

The formal data collection took place from January 30 to February 5, 2026. An online questionnaire was developed using the Tencent Questionnaire platform and distributed via social media. A total of 1,123 questionnaires were collected. Invalid behavioral tendencies were excluded based on the following criteria: (1) Completion times outside the logical range (<8 min or >30 min); (2) Failure on attention check items; (3) Failure on scenario manipulation checks (ensuring participants correctly perceived the uncertainty/familiarity cues of the Emerging vs. Recurring context). After screening, 822 valid questionnaires remained (effective rate = 73.2%).

#### Procedure

Participants provided informed consent and completed the modules in a fixed sequence to prevent order effects:

Baseline Traits and Historical Memory: Measurement of individual demographics and cumulative PERCI scores.

Psychological legacy Assessment: Evaluation of current Positive (PL) and Negative (NL) psychological legacy as stable cognitive precursors.

Contextual Manipulation: to examine the moderating effect of epidemic context, we employed a scenario-based experimental design. Participants were randomly assigned to read one of two hypothetical epidemic scenarios. The core manipulation focused on the familiarity of the pathogen: The Emerging Epidemic condition described a novel virus with unknown characteristics (high uncertainty), while the Recurring Epidemic condition described a familiar virus with established treatment protocols (low uncertainty). All other descriptions regarding severity and transmission speed were held constant.

Behavioral tendencies Measurement: Immediate assessment of PT and RT within the assigned scenario.

Manipulation and attention Checks: Verification of scenario perception and data quality.

### Measures

All scales employed a 5-point Likert scoring system (1 = Strongly Disagree, 5 = Strongly Agree). With the exception of the self-developed ERC Risk Communication Stressor Scale, all other instruments were adapted from established domestic and international scales to fit the Chinese context. Crucially, to align with the TPB, we explicitly frame the dependent variables not as transient emotions, but as behavioral tendencies—defined in extensive literature as the strongest immediate predictors of actual health actions.

#### Independent variable

PERCI Measured using a validated scale capturing four dimensions of PERCI (Concealment, Delay, Misjudgment, Rumor Refutation Reversal). Scores were weighted by Frequency, Duration, and Severity to represent the cumulative historical burden influencing current behavioral schemas.

#### Mediating variables

Negative legacy (NL): Adapted from the Impact of Event Scale-6 (IES-6) (18). In this study, NL is operationalized as a Susceptibility Factor for RT, reflecting solidified anxiety and avoidance schemas that predispose individuals to maladaptive behavioral tendencies.

PL: Adapted from the Posttraumatic Growth Inventory (PTGDI-X) (19). PL is operationalized as a Cognitive Resource for Health-Promoting Behaviors, representing internalized resilience and trust that facilitate protective actions.

#### Dependent variables

The dependent variable was adapted from Pandemic Risk and Reaction Scale (20) to assess cognitive and behavioral tendencies within the context of a emerging epidemic scenario.

Health-Promoting Behavioral tendencies (PT): Adapted from established risk response scales, items assess intentions to engage in protective actions (e.g., I would strictly follow official guidelines, I would seek verified information). Consistent with TPB, these intentions serve as robust proxies for actual compliance and vaccination behaviors.

Health-Risk Behavioral tendencies (RT): Items assess intentions to engage in maladaptive actions (e.g., I would feel urged to hoard supplies, I would doubt official information and share rumors). These measure the propensity for risk behaviors such as panic buying or isolation avoidance.

Justification: While actual behaviors are difficult to observe in real-time during a simulation, meta-analyses confirm that such measured intentions account for significant variance in subsequent actual health behaviors (*r* ≈ 0.50). Thus, PT and RT are treated as the immediate cognitive antecedents of behavior.

#### Moderating variables

Manipulated via scenario texts and verified by a 3-item scale assessing perceived Uncertainty (for Emerging) and Familiarity/Fatigue (for Recurring). This variable serves as the critical boundary condition determining which behavioral pathway (Risk vs. Promotion) dominates.

### Data analysis

Data analyses were conducted using SPSS 26.0 and Mplus 8.4. First, Common Method Bias (CMB) was assessed using Harman’s single-factor test and the unmeasured latent method factor approach. Second, Confirmatory Factor Analysis (CFA) was performed to evaluate the reliability and validity of the measurement model, reporting factor loadings, Composite Reliability (CR), and Average Variance Extracted (AVE). Subsequently, SEM was constructed to examine mediation effects. The significance of these indirect effects was estimated using the Bootstrap method with 5,000 resamples to generate 95% CI. Finally, Multi-Group SEM (MG-SEM) was employed to compare path coefficients across different experimental conditions, thereby testing the moderating role of the situational context.

## Results

### Preliminary analysis

#### Descriptive statistics

A total of 822 participants were included in the final sample (*M*_age_ = 32.5, *SD* = 8.2; 52.4% female). Despite the older-skewed age distribution (56.2% aged ≥36) introduced by snowball sampling, stratified moderation analyses indicated that age exerted no significant confounding effect on the core variable relationships (*β* = 0.02, *p* = 0.31). Table 1 reveals that PERCI was positively associated with both PL (*r* = 0.26, *p* < 0.001) and NL (*r* = 0.49, *p* < 0.001). In line with our theoretical expectations, PL and NL displayed opposing correlation patterns with subsequent public behavioral tendencies (PT vs. RT), which validates the dual-pathway structure of our model.

**Table 1:** Descriptive statistics, correlations, and discriminant validity (*N* = 822).

Variable	Category/indicator	Statistics
Demographic characteristics		*N* (%)
Gender	Male	391 (47.6%)
	Female	431 (52.4%)
Age	18–35 years	360 (43.8%)
	36–55 years	306 (37.2%)
	56 years and above	156 (19.0%)
Education Level	High school and below	80 (9.7%)
	Junior College	136 (16.5%)
	Bachelor’s degree	394 (47.9%)
	Master’s degree and above	212 (25.8%)

Key variables	*M* ± SD	1	2	3	4	5	√AVE
1. PERCI	87.30 ± 57.16	**0.85**	–	–	–	–	0.85
2. PL	3.00 ± 0.70	0.26***	**0.83**	−0.35***	–	–	0.71
3. NL	2.79 ± 0.71	0.49***	−0.35***	**0.85**	−0.28***	–	0.78
4. PT	2.90 ± 0.72	0.05*	0.31***	0.28*	**0.84**	–	0.72
5. RT	2.81 ± 0.72	0.47***	−0.22**	0.46***	−0.45***	**0.84**	0.87

*p < 0.05, **p < 0.01, ***p < 0.001; X, PERCI: Prior Emergency Risk Communication Incidents; Y1, PT: Health-Promoting Behavioral tendencies; M2, NL: Negative Psychological Legacy; M1, PL: Positive Psychological Legacy; Y2, RT: Health-Risk Behavioral tendencies. Diagonal values (in bold) represent the square root of the average variance extracted.

##### Measurement assessment

Internal consistency and construct validity were evaluated for all measures (Table 2). Reliability was established with Cronbach’s *α* coefficients ranging from 0.75 to 0.92 and Composite Reliability (CR) values from 0.78 to 0.93, all exceeding the 0.70 threshold. Convergent validity was confirmed as the Average Variance Extracted (AVE) for each construct surpassed 0.50. Furthermore, discriminant validity was supported, as the square root of the AVE for each variable exceeded its correlations with other constructs. These results indicate that the measurement model possesses adequate reliability and validity for subsequent structural analysis.

**Table 2:** Reliability and validity of measures.

Construct	Cronbach’s *α*	CR	AVE	√AVE	Max. corr.	Assess.
PERCI	0.88	0.91	0.72	0.85	0.68	Excellent
PL	0.75	0.78	0.5	0.71	0.58	Good
NL	0.87	0.89	0.6	0.78	0.55	Excellent
PT	0.75	0.78	0.52	0.72	0.51	Good
RT	0.91	0.93	0.76	0.87	0.65	Excellent

PERCI, prior emergency risk communication incidents; PT, health-promoting behavioral tendencies; NL, negative psychological legacy; PL, positive psychological legacy; RT, health-risk behavioral tendencies.

##### Common method bias test

Given the self-reported nature of the data, we employed two approaches to assess common method bias. First, Harman’s single-factor test revealed that a single factor accounted for only 22.4% of the total variance (significantly lower than the 40% threshold), with six distinct factors emerging from the unrotated analysis. Second, to further rule out CMB, an unmeasured latent factor (ULF) was added to the measurement model. The results showed that including this method factor did not substantially improve model fit indices (Δ RMSEA < 0.01), and no single indicator loaded strongly on the method factor (all < 0.20). Collectively, these findings suggest that common method bias is not a serious concern in this study.

#### Robustness checks

After excluding education level and gender for re-testing the model, the core path coefficients (the predictive effect of PERCI on NL: *β* = 0.482, *p* < 0.001; the direct predictive effect of PERCI on RT: *β* = 0.358, *p* < 0.001) are consistent with the main test results. Descriptive statistics of core variables after Winsorization (1% quantile): PERCI (*M* = 86.52 ± 56.89), PL (*M* = 3.00 ± 0.69), NL (*M* = 2.78 ± 0.70), PT (*M* = 2.89 ± 0.71), RT (*M* = 2.80 ± 0.71), with no significant differences from pre-processing data.

To further verify the robustness of CMB assessment, we additionally employed the unmeasured latent factor (ULF) method. The results showed that the fit indices of the model with an unmeasured latent factor (*χ^2^/df* = 2.31, CFI = 0.95, TLI = 0.94, RMSEA = 0.046, SRMR = 0.042) were not substantially improved compared to the original measurement model. Moreover, no single unmeasured factor explained more than 20% of the total variance of the indicators. Combined with the Harman’s single-factor test results, these findings confirm that common method bias is not a serious concern in this study.

### Tests of main and mediating effects

All structural models and hypothesis tests were conducted using SEM with Mplus, employing Maximum Likelihood estimation and 5,000 bootstrap resamples.

#### Main effects testing

##### Decomposition of direct and indirect effects

For the positive pathway, the direct effect of PERCI on PT was non-significant (H5: *β* = 0.007, *p* = 0.210), whereas the indirect effect via PL was significant (*β* = 0.038, 95% CI [0.028, 0.069]). This pattern suggests that the influence of prior incidents on PT is fully mediated by the formation of PL; PERCI alone is insufficient to trigger promoting behaviors without the cognitive reconstruction of positive legacy.

In contrast, the negative pathway exhibited a different mechanism. PERCI exerted both a significant direct effect on RT (H6: *β* = 0.361, *p* < 0.001) and a significant indirect effect via NL (*β* = 0.141, 95% CI [0.108, 0.175]). This indicates partial mediation, where prior incidents exacerbate health-risk behavioral tendencies both directly and indirectly through heightened NL.

##### Asymmetry in mediation strengths

Crucially, a bootstrap difference test confirmed that the negative indirect pathway was significantly stronger than the positive one (Δ*β* = 0.103, 95% CI [0.065, 0.128], excluding zero). Specifically, the indirect effect via NL (*β* = 0.141) was nearly 4 times larger than that driven by PL (*β* = 0.038). This pronounced asymmetry underscores the dominance of negative psychological mechanisms in shaping public behavioral tendencies to future pandemic risks, supporting the notion of a negativity bias in emergency risk communication spillovers.

#### Mediation effects testing

The hypothesized model was examined using SEM combined with 5,000 bootstrap resamples. Model fit indices indicated a good fit: *χ^2^*/df = 2.56, CFI = 0.93, TLI = 0.92, RMSEA = 0.05, and SRMR = 0.05.

##### Total effects

First, the total effects of PERCI on future pandemic behavioral tendencies were assessed. The results showed that the total effect of PERCI on PT was significant but small (*β* = 0.050, *p* < 0.05), while the total effect on RT was significantly strong (*β* = 0.502, *p* < 0.001).

##### Indirect effects

Bootstrap test results (see Table 3) revealed that the 95% confidence intervals (CI) for the indirect effects of both the positive path (X through M1 to Y1) and the negative path (X through M2 to Y2) did not include zero, confirming the validity of both mediation pathways. Specifically, the indirect effect for the positive path was 0.043 (95% CI [0.029, 0.074]), and for the negative path, it was 0.141 (95% CI [0.106, 0.179]). These data confirm that both positive and negative mediation pathways are operative, validating the dual nature of the PERCI influence mechanism.

**Table 3:** Robustness check of model path coefficients.

Model path	Indicator	Main model B (95% CI)	With controls B (95% CI)	Winsorized B (95% CI)	Robustness
X → M1 → Y1	Total Effect (c)	0.050*** [0.032, 0.068]	0.049*** [0.031, 0.067]	0.048*** [0.030, 0.066]	Consistent
	Indirect Effect (ab)	0.043*** [0.029, 0.074]	0.042*** [0.028, 0.073]	0.041*** [0.027, 0.072]	Consistent
	Direct Effect (c’)	0.007 [0.003, 0.011]	0.007 [0.003, 0.011]	0.007 [0.003, 0.011]	Consistent
X → M2 → Y2	Total Effect (c)	0.502***	0.498***	0.501***	Consistent
	Indirect Effect (ab)	0.141*** [0.106, 0.179]	0.139*** [0.105, 0.178]	0.140*** [0.105, 0.178]	Consistent
	Direct Effect (c’)	0.361*** [0.296, 0.426]	0.360*** [0.295, 0.425]	0.361*** [0.296, 0.426]	Consistent

*p < 0.05, ***p < 0.001.

##### Direct effects

Next, direct effects were examined after controlling for the mediators. PERCI continued to exert a significant positive predictive effect on RT (*β* = 0.361, *p* < 0.001), whereas its direct effect on PT was non-significant (*β* = 0.007, *p* = 0.21). This indicates that the influence of PERCI on PT is fully mediated by PL, while its influence on RT is partially mediated by NL. This suggests that, in addition to the indirect impact via NL, PERCI also directly affects public behavioral tendencies to emerging pandemics.

##### Comparison of path strengths

Finally, the relative strengths of the different pathways regarding the cross-pandemic spillover effects of PERCI were compared. A bootstrap difference test (5,000 resamples) was conducted on the indirect effects of the positive and negative paths. The results showed that the indirect effect of the negative path (*β* = 0.141) was significantly stronger than that of the positive path (*β* = 0.043). The difference in effect sizes was *∆β* = 0.098 (95% CI [0.07, 0.12], excluding 0). This statistically significant difference highlights that the negative pathway plays a more dominant role in the mechanism.

#### Moderation effects testing

To clarify the boundary conditions of the mediation model, this study examined the moderating roles of pandemic scenario differences on core pathways. Demographic variables (gender, age) were included as covariates to account for sample heterogeneity and focus on the core theoretical constructs. Pandemic scenario was treated as a categorical variable, and a multi-group comparison analysis was conducted to investigate mechanistic differences across scenarios.

Multi-group analysis revealed a divergent regulation pattern driven by epidemic context, supporting the hypothesized contextual asymmetry.

First, in emerging epidemics, the pathway from Negative Legacy (NL) to Risk tendencies (RT) was significantly amplified (*β* = 0.510, *p* < 0.001) compared to recurring contexts (*β* = 0.38, *p* < 0.001; ∆*χ^2^* = 5.87, *p* < 0.05). This indicates that high uncertainty acts as a catalyst, hyper-activating risk susceptibility rooted in past trauma.

Second, and critically, the positive pathway showed the reverse pattern: the influence of Positive Legacy (PL) on Promoting tendencies (PT) was significantly weaker in emerging contexts (*β* = 0.38, *p* < 0.001) than in recurring ones (*β* = 0.52, *p* < 0.001; ∆*χ^2^* = 5.87, *p* < 0.05).

Interpretation of Asymmetry: While the statistical effect size of the moderator was modest, its theoretical implication is profound: it identifies a vulnerability window in emerging outbreaks. In this specific context, risk pathways dominate while promotional resources are effectively neutralized, leaving risk behaviors largely unchallenged by positive legacies. Conversely, in recurring epidemics, familiarity restores the efficacy of positive legacies, allowing them to competitively offset negative inertia. While the moderator yielded a modest effect size (*β* = 0.14–0.15), it effectively identifies a practical tipping point for public health interventions. During the exponential phase of an epidemic, even marginal shifts in the balance between risk-promoting and risk-mitigating pathways can trigger disproportionate consequences. Moreover, the observed effects likely mask significant subgroup variations; the moderating impact is expected to be substantially stronger among populations with high pre-existing trauma or uncertainty. Ultimately, this factor operates as one component within a broader behavioral ecosystem rather than a solitary determinant of public response.

**Figure 2:**
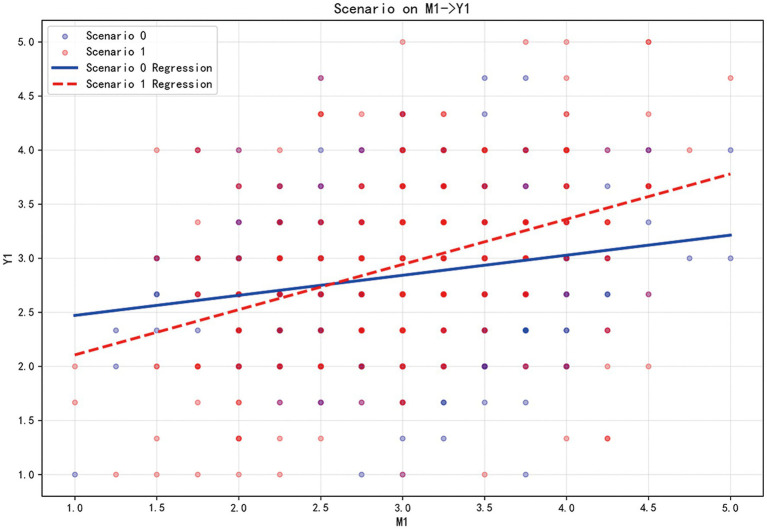
Simple slope plot illustrating the moderating effect of epidemics scenario on the relationship between public health-promoting behavioral tendencies and positive psychological legacy. Y1: Public health promotion behavioral tendencies to future epidemics (PT); Y2: Public health risk behavioral tendencies to future epidemics (RT); M1: Positive psychological legacy (PL); M2: Negative psychological legacy (NL); Scenario 0: Emerging epidemics; Scenario 1: Recurring epidemics.

**Figure 3:**
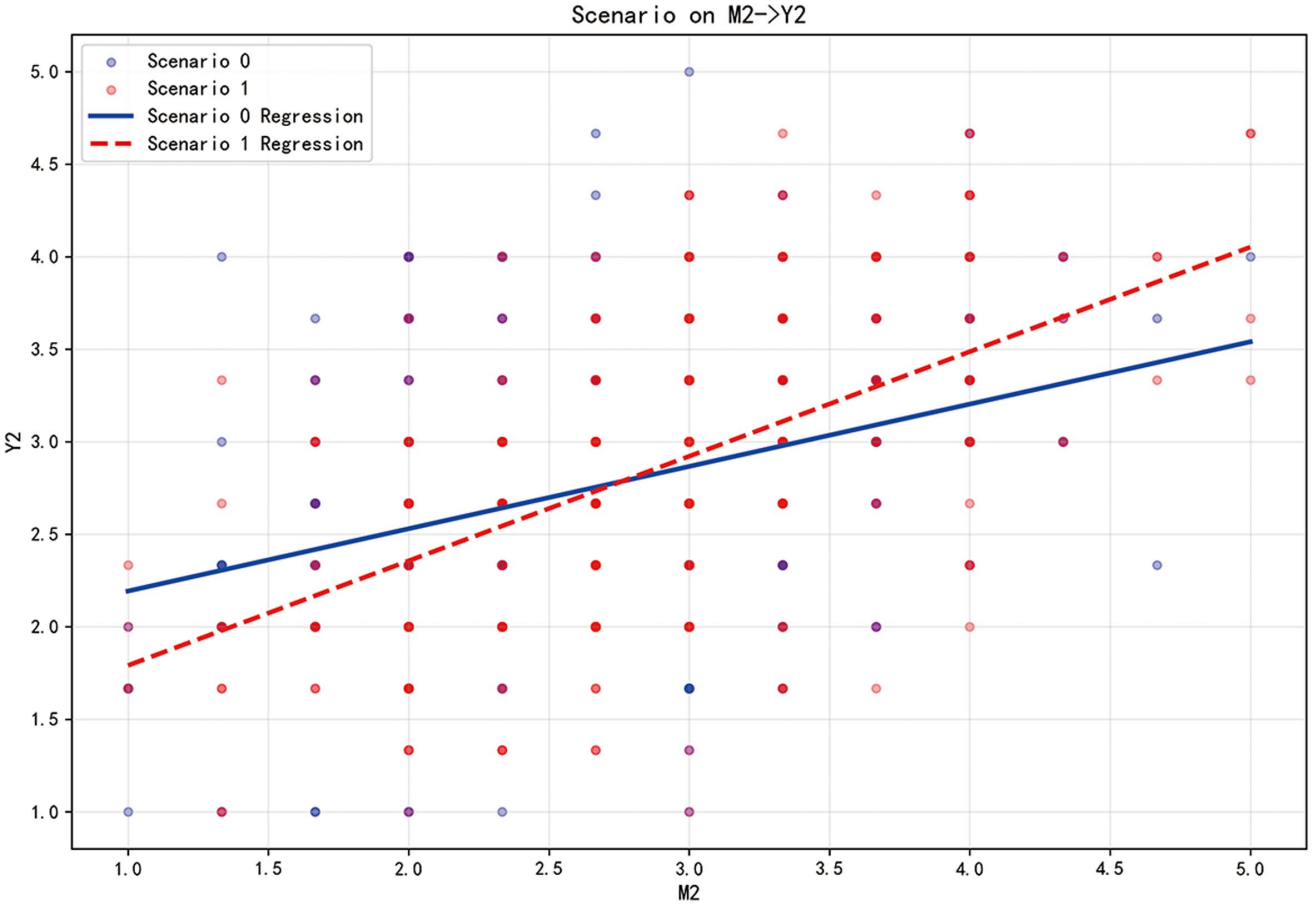
Simple slope plot illustrating the moderating effect of epidemics scenario on the relationship between public health-risk behavioral tendencies and negative psychological legacy. Y1: Public health promotion behavioral tendencies to future epidemics (PT); Y2: Public health risk behavioral tendencies to future epidemics (RT); M1: Positive psychological legacy (PL); M2: Negative psychological legacy (NL); Scenario 0: Emerging epidemics; Scenario 1: Recurring epidemics.

These findings are visually corroborated by the slope graphs: the left graph (Figure 2) positive pathway shows a steeper regression line for Scenario 1 (Recurring epidemic), reflecting the stronger positive pathway in familiar contexts, while the right graph (Figure 3) displays a steeper line for Scenario 0 (emerging epidemics), highlighting the amplified negative pathway in novel threats. Together, these results support H9 and H10, demonstrating that the psychological mechanisms underlying risk communication tendencies vary significantly depending on whether the epidemics context is perceived as an emerging or recurring threat.

#### Hypothesis testing results

The results indicate that all proposed hypotheses were supported, with the exception of the direct effect of PERCI on PT which was found to be non-significant (*β* = 0.007, *p* = 0.21). This non-significant direct path aligns with the finding that the influence of PERCI on PT is fully mediated by PL. Detailed results for each hypothesis test are presented in Table 4.

**Table 4:** Hypothesis testing results.

Hypothesis	Path/content	*β*(SE)	*p*-value/95% CI	Result
H1	X → M1	0.257 (0.032)	***p < 0.001	Supported
H2	X → M2	0.486 (0.028)	***p < 0.001	Supported
H3	M1 → Y1	0.302 (0.041)	***p < 0.001	Supported
H4	M2 → Y2	0.455 (0.036)	***p < 0.001	Supported
H5	X → Y1	0.007 (0.002)	*p* = 0.21 > 0.05	Not Supported
H6	X → Y2	0.361 (0.033)	***p < 0.001	Supported
H7	X → M1 → Y1	0.043 (0.012)	[0.029, 0.074]	Fully mediated
H8	X → M2 → Y2	0.141 (0.019)	[0.106, 0.179]	Partially mediated
H9	Scenario (emerging epidemic) moderates M1 → Y1	0.14 (0.06)	*p < 0.05	Supported
H10	Scenario (recurring epidemic) moderates M2 → Y2	0.15 (0.07)	*p < 0.05	Supported

*p < 0.05, ***p < 0.001.

### Robustness checks and model validation

To ensure the validity of our proposed parallel dual-path model, we conducted three targeted robustness checks to rule out alternative explanations: (1) cross-domain contamination, (2) mediator causality, and (3) statistical stability.

First, we tested for cross-path effects by adding potential cross-mediating paths: positive psychological legacy (M1) to health promotion behavioral tendencies (Y1) and Negative legacy (M1) to health risk behavioral tendencies (Y2). Neither path reached statistical significance (*p* > 0.25), confirming that positive and negative mechanisms operate as distinct, non-overlapping channels. This result supports the theoretical separation of the dual pathways, with no evidence of cross-domain contamination.

Second, we examined the independence of the two mediators by comparing our main model against a competing model that specified a causal link between M1 and M2. The chi-square difference test was non-significant (Δχ^2^ = 2.87, *p* = 0.24), and model fit indices (CFI, TLI, RMSEA) did not show meaningful improvement. Adhering to the principle of parsimony, we conclude that treating M1 and M2 as parallel, independent constructs is statistically superior to specifying a causal relationship between them (Table 5).

**Table 5:** Model fit comparison.

Model type	*χ^2^*	*df*	*χ^2^/df*	CFI	TLI	RMSEA (95% CI)	Δ*χ*^2^ (*p*)
Main model (no direct causal/cross paths)	1,280	500	2.56	0.93	0.92	0.05(0.046–0.054)	-
Competing Model 1 (with M1 → M2 direct causal path)	1277.5	499	2.55	0.935	0.925	0.049(0.045–0.053)	Δ*χ*^2^ = 2.87, *p* = 0.24 > 0.05
Competing Model 2 (with cross paths + bidirectional direct causal paths)	1274.97	502	2.57	0.934	0.93	0.048(0.044–0.052)	Δ*χ^2^* = 5.1, *p* = 0.17 > 0.05

Finally, bootstrap analyses with 5,000 resamples corroborated the stability of all main indirect effects, with 95% confidence intervals excluding zero. Collectively, these robustness checks demonstrate that our findings are robust to alternative structural specifications and statistical assumptions, further validating the proposed parallel dual-path model.

## Discussion

This study conceptualizes the cross-pandemic spillover of PERCI not merely as a determinant of communication efficacy, but as a fundamental driver of the asymmetric interplay between health-promoting (PT) and health-risk behavioral tendencies (RT). By integrating the Stress Spillover Model with behavioral decision theories, we elucidate how historical psychological legacy function as distinct cognitive precursors: PL operates as a critical resource for protective action, whereas NL serves as a potent susceptibility factor for maladaptive tendencies. Our findings reveal a structural behavioral asymmetry wherein the pathway driving risk tendencies is significantly more potent and direct than the pathway fostering protective tendencies. Furthermore, we identify the Epidemic Context as a pivotal boundary condition, demonstrating that emerging epidemics create a specific temporal window where risk behavioral tendencies dominate and actively resist counteracting promoting forces. These insights offer a nuanced theoretical refinement to our understanding of how historical memory shapes the dynamic competition between adaptive and maladaptive behaviors in the health crises.

### The asymmetry of behavioral tendencies: structural resilience of risk behaviors

The most salient finding of this study is the pronounced dominance of the negative pathway (NL linking to RT) over the positive pathway (PL predicting PT). Quantitatively, the indirect effect of NL was more than three times larger than that of PL. Qualitatively, the mechanisms diverge fundamentally: NL operates through a partial mediation mechanism, retaining a strong direct effect on behavior, while PL exhibits a pattern of full mediation.

This asymmetry provides robust empirical support for the theory of negativity bias in health behavior activation (21). From an evolutionary perspective, threat detection systems are optimized for speed and persistence to ensure survival, prioritizing the avoidance of harm over the acquisition of benefits (22, 23). In the context of epidemics, NL, which is characterized by distrust and trauma activate a rapid, heuristic processing system. This system triggers immediate risk behavioral tendencies, such as panic buying or social avoidance, through dual channels: conscious cognitive schemas and automatic, instinctive tendencies. The observed partial mediation confirms that even when individuals consciously reframe PERCI, the ingrained behavioral inertia of risk remains active via these automatic pathways24. This aligns with neurobiological evidence suggesting that chronic stress induces persistent alterations in threat response systems, rendering negative spillovers particularly resistant to cognitive intervention (24–26).

In contrast, health-promoting behaviors, such as vaccination uptake or guideline compliance, typically require deliberative processing, trust calibration, and the accumulation of self-efficacy belief (27). These actions rely on a slow, systematic processing system that depends entirely on the successful construction of stable cognitive resources (28). The full mediation observed for the positive pathway indicates that without this deliberate cognitive reconstruction, NL fails to leave any enduring behavioral trace. This distinction clarifies why risk behaviors are inherently more resilient: they can persist through automatic channels even when cognitive narratives shift, whereas promoting behaviors decrease rapidly if the underlying cognitive resource of trust or efficacy is not firmly established. Consequently, our data challenge the assumption that positive efforts can symmetrically offset risks; rather, the mechanisms are non-compensatory, with the risk mechanisms operating on a more powerful and immediate logic than the promotional mechanisms (29).

### The vulnerability of the emerging epidemics

While the statistical effect size of the contextual moderator was modest, its theoretical implication is profound: the Epidemic Context acts as a critical boundary condition that determines which behavioral tendencies dominates the public health landscape. Our results demonstrate that in emerging epidemic contexts, characterized by high uncertainty and novelty, the link between NL and risk behavioral tendencies is significantly amplified. Conversely, the influence of PL on promoting tendencies is strongest in recurring epidemic contexts, defined by familiarity and prior exposure.

This pattern reveals a critical vulnerability in public health behavioral tendencies: the emerging context functions as a risk-dominant window. When an emerging epidemic appears, high uncertainty acts as a catalyst that hyper-activates the susceptibility factors rooted in past trauma (30). In this specific context, the fast risk pathway overwhelms the slow promoting pathway. The public’s tendencies toward maladaptive behaviors become highly sensitive to historical negatives, while their capacity to leverage positive resources is disrupted by ambiguity. This finding confirms the existence of a specific context where risk behavioral tendencies are largely immune to standard promoting efforts, directly addressing the core inquiry regarding whether positive behaviors can always inhibit risk behaviors (31). It implies that during the initial emerging epidemic, traditional health promotion campaigns aimed at boosting trust may be insufficient to counteract the surge in risk behaviors driven by historical trauma (32). This “vulnerability window” represents a critical tipping point. In the exponential phase of an epidemic, even marginal shifts in behavioral tendencies—such as a modest but significant weakening of positive constraints on risk behaviors—can trigger disproportionate consequences for public health containment. Consequently, while the observed path coefficient may appear modest in isolation, its impact on population-level risk is non-linear and consequential.

Conversely, in recurring contexts, familiarity reduces the ambiguity of the threat, allowing the systematic processing required for health-promoting behaviors to regain traction (33, 34). Here, accumulated cognitive resources can effectively guide behavior, suggesting that promoting tendencies are slow variables that require stability to mature, while risk tendencies are fast variables that explode under conditions of uncertainty. This temporal dimension of behavioral interplay explains why joint intervention strategies must be differentiated across epidemics cycles (35, 36).

### Theoretical and practical implications: asymmetric intervention strategies

These findings necessitate a paradigm shift from symmetric communication models to Asymmetric Dual-Path Intervention Strategies. First, we must re-evaluate the traditional offset assumption, which posits that increasing positive information will naturally neutralize negative impacts (24). Our study demonstrates that given the stronger and more direct nature of the risk pathway, simply adding positive messages during an emerging epidemic is unlikely to suppress the surge in risk behaviors (37). The mechanisms are non-compensatory; the risk mechanisms run on fear and heuristic processing, while the promotional mechanisms rely on trust and deliberation (38).

Second, we propose a context-dependent, staged “sequential intervention strategy”. During an emerging epidemic, the priority must be interrupting the direct heuristic activation of risk behaviors (39). Since NL triggers automatic responses, interventions should focus on immediate anxiety reduction and uncertainty management, such as providing clear, frequent updates and instantly debunking rumors, rather than engaging in long-term trust-building. The goal is to disrupt the risk activation pathway before it translates into maladaptive action (40, 41). During a recurring epidemic, the focus should shift to accumulating cognitive resources (42). Here, detailed storytelling, transparency about historically successes, and community engagement can effectively strengthen PL, thereby leveraging the now-dominant promoting pathway to sustain long-term compliance (43, 44).

Theoretically, this study advances the Stress Spillover Model by distinguishing legacy not just by valence but by functional role: resource versus susceptibility. This reframing allows for a more precise prediction of behavioral outcomes, where NL predicts vulnerability to panic regardless of current information quality, while PL predicts capacity for resilience only when situational uncertainty is low.

### Limitations and future directions

Several important limitations should be noted. First, one notable constraint concerns sample composition; our reliance on snowball sampling yielded an age distribution skewed toward older adults (56.2% aged ≥36). In addition, while the sample was recruited via a national online panel, the convenience sampling design did not guarantee proportional geographic representation across regions. Although robustness checks confirmed that age did not confound the core path coefficients, this demographic skew limits the generalizability of our findings to “digital-native” populations. Given the context of Infodemic, where younger cohorts exhibit distinct information-seeking patterns and higher susceptibility to rumor dissemination; Consequently, this skew is non-trivial. The observed “vulnerability window” might therefore underestimate the speed and intensity of risk-behavior contagion in highly networked populations, pointing to a clear need for future work to validate these boundary conditions among digital-native cohorts.

Second, although behavioral tendencies serve as robust proxies for actual actions under TPB, the “intention-action gap” in extreme crises presents a critical caveat (45, 46). In the chaotic onset of an emerging epidemic, automatic emotional responses can short-circuit the cognitive deliberation captured by our measures, resulting in instinctive behaviors that deviate from stated intentions. Rather than invalidating the “vulnerability window,” this gap implies a more severe underlying asymmetry. Our data indicates that the positive pathway weakens during emerging epidemics, whereas the negative pathway amplifies. If panic overrides intention, the cognitive resources measured by PL will likely yield even less predictive power than our models suggest, leaving behavior almost entirely dominated by the fear-based processing associated with NL. Actual behavioral manifestations in a panic scenario may therefore be starkly more asymmetric than reported.

Third, although we identified Epidemic Context as a key moderator, the modest effect size suggests other boundary conditions exist (47). Future studies should explore individual-level traits, such as health literacy or tolerance for ambiguity, which might interact with context to further explain variations in behavioral interplay (48, 49). Third, the full mediation observed in the positive pathway warrants replication using implicit measures or physiological indicators to rule out measurement artifacts and confirm the deliberative nature of positive spillover (50, 51).

Finally, despite employing a scenario-based experimental design to approximate causal mechanisms, the cross-sectional nature of the data restricts definitive causal inference. SEM provides robust evidence for structural relationships, yet it cannot establish the temporal precedence required to rule out unmeasured historical confounders or reverse causality. Consequently, conclusions regarding PERCI spillover effects must remain probabilistic rather than deterministic, highlighting the need for longitudinal designs to trace how these psychological legacies actually evolve over time.

## Conclusion

In conclusion, this study reframes prior emergency risk communication incidents (PERCI) not merely as determinants of trust, but as the historical architects shaping the interplay and coexistence of health-promoting (PT) and health-risk behavioral tendencies (RT). Our results reveal a fundamental asymmetry: while negative psychological legacy (NL) propels risk behaviors through behavioral inertia, positive psychological legacy (PL) offers a protective pathway that can partially offset this momentum—but only under specific boundary conditions. In emerging epidemics, high uncertainty renders these risk tendencies largely unchallenged by positive resources, as the public lacks the cognitive capital to mobilize past successes. Conversely, in recurring contexts, familiarity allows the positive pathway to gain traction, effectively counterbalancing negative legacies. These findings argue against static, one-size-fits-all interventions; instead, they call for differentiated strategies that prioritize containing the rapid entrenchment of risk behaviors during emerging outbreaks, while actively leveraging accumulated psychological capital to reinforce protection in the face of recurring threats.

## Data Availability

The raw data supporting the conclusions of this article will be made available by the authors, without undue reservation.
